# Macrophage and T Cell Produced IL-10 Promotes Viral Chronicity

**DOI:** 10.1371/journal.ppat.1003735

**Published:** 2013-11-07

**Authors:** Kirsten Richter, Guillaume Perriard, Rayk Behrendt, Reto A. Schwendener, Veronika Sexl, Robert Dunn, Masahito Kamanaka, Richard A. Flavell, Axel Roers, Annette Oxenius

**Affiliations:** 1 Institute of Microbiology, ETH Zurich, Zurich, Switzerland; 2 Institute of Immunology, Technical University of Dresden, Dresden, Germany; 3 Institute of Molecular Cancer Research, University of Zurich, Zurich, Switzerland; 4 Institute of Pharmacology and Toxicology, University of Veterinary Medicine Vienna,Vienna, Austria; 5 Biogen Idec, San Diego, California, United States of America; 6 Department of Immunobiology and Howard Hughes Medical Institute, Yale School of Medicine, New Haven, Connecticut, United States of America; University of Pennsylvania, United States of America

## Abstract

Chronic viral infections lead to CD8^+^ T cell exhaustion, characterized by impaired cytokine secretion. Presence of the immune-regulatory cytokine IL-10 promotes chronicity of Lymphocytic Choriomeningitis Virus (LCMV) Clone 13 infection, while absence of IL-10/IL-10R signaling early during infection results in viral clearance and higher percentages and numbers of antiviral, cytokine producing T cells. IL-10 is produced by several cell types during LCMV infection but it is currently unclear which cellular sources are responsible for induction of viral chronicity. Here, we demonstrate that although dendritic cells produce IL-10 and overall IL-10 mRNA levels decrease significantly in absence of CD11c^+^ cells, absence of IL-10 produced by CD11c^+^ cells failed to improve the LCMV-specific T cell response and control of LCMV infection. Similarly, NK cell specific IL-10 deficiency had no positive impact on the LCMV-specific T cell response or viral control, even though high percentages of NK cells produced IL-10 at early time points after infection. Interestingly, we found markedly improved T cell responses and clearance of normally chronic LCMV Clone 13 infection when either myeloid cells or T cells lacked IL-10 production and mice depleted of monocytes/macrophages or CD4^+^ T cells exhibited reduced overall levels of IL-10 mRNA. These data suggest that the decision whether LCMV infection becomes chronic or can be cleared critically depends on early CD4^+^ T cell and monocyte/macrophage produced IL-10.

## Author Summary

Chronic viral infections like Hepatitis B and C Virus (HBV and HCV) and Human Immunodeficiency Virus (HIV) in humans affect more than 500 million people worldwide. While a robust T cell response is a hallmark of many acute infections one hurdle inhibiting the clearance of chronic viral infections is that the immune-suppressive cytokine IL-10 modulates the virus-host balance towards induction of T cell dysfunction. IL-10 is produced by several cell types during chronic Lymphocytic Choriomeningitis Virus (LCMV) infection but it is currently unclear which cellular sources are responsible to promote viral chronicity. Here, we demonstrate that T cell responses improved markedly, and that normally chronic LCMV Clone 13 infection could be cleared when either myeloid cells or T cells lacked IL-10 production. Furthermore, mice depleted of monocytes/macrophages or CD4^+^ T cells exhibited reduced overall levels of IL-10 mRNA. These data suggest that the decision whether LCMV infection becomes chronic or can be cleared critically depends on CD4^+^ T cell and monocyte/macrophage produced IL-10 early during the establishment of viral chronicity.

## Introduction

The functional down regulation of antiviral T cells, also termed T cell exhaustion, is a major hurdle inhibiting the control or even clearance of chronic infections. T cell exhaustion is characterized by a gradual loss of cytokine producing antiviral CD8^+^ T cells [1]. The host-derived anti-inflammatory cytokine IL-10 is an important player in driving T cell exhaustion and viral chronicity in LCMV Clone 13 infected mice [2], [3], a commonly used murine model for chronic viral infections. Elevated IL-10 levels were also found to correlate with HIV replication in humans and transition to chronicity during HBV and HCV infection [4], [5], [6]. Since disruption of IL-10 receptor signaling enhances CD8^+^ T cell effector functions not only after LCMV infection [2], [3], but also in case of HIV-, HBV- or HCV-specific T cells [6], [7], [8], [9], interference with IL-10 signaling is currently proposed as a target for immune-based interventions during chronic viral infections.

IL-10 is expressed during several persistent infections. It may on the one hand favor viral chronicity by suppressing the antiviral immune response, but on the other hand also protect the host from immunopathology [10], [11]. IL-10 acts mainly by modulating the expression of proinflammatory cytokines and chemokines, by modulating the function of antigen presenting cells (i.e. down-regulating for example the expression of MHCI, MHCII, B7-1 and B7-2) and by directly or indirectly suppressing proliferation, functional differentiation and effector activity of antiviral T cells [10], [11]. Suppression of the antiviral immune response through IL-10 is a strategy actively exploited by herpes- and poxviruses which can encode viral IL-10 homologues to weaken the antiviral immune response [12]. Accordingly, rhesus macaques infected with rhesus CMV deficient for rhcmvIL-10 exhibit a T and B cell response of higher quality and quantity [13].

In addition, genetic polymorphisms in the IL-10 promoter are associated with decreased IL-10 production, leading to enhanced control of HCV, HBV, HIV and Epstein Barr Virus (EBV) [14], [15], [16], [17], [18]. Despite the advantages of more effective immune responses in absence of IL-10, this may come at the expense of immunopathology [19], [20], [21]. It is currently unclear which cellular source(s) of IL-10 are decisive in promoting chronicity of high dose LCMV infection. Several cell populations have been reported to produce IL-10 after LCMV infection: LCMV specific CD4^+^ T cells secrete IL-10 on day 5 post infection [22], IL-10 mRNA is upregulated in dendritic cells (DCs), B cells, macrophages, CD4^+^ and CD8^+^ T cells on day 9 post infection [2] and purified CD4^+^ T cells and DCs produce IL-10 upon LCMV infection of splenocytes *in vitro* [3]. Furthermore, high percentages of DCs and macrophages, but also B cells, CD4^+^ and CD8^+^ T cells produce IL-10 nine and thirty days after Clone 13 infection when assessed in IL-10 reporter (VertX) mice [23]. Significant IL-10 production was found mainly in CD8^−^ DCs and IL-10 serum protein levels were decreased in mice with selective IL-10 deficiency in CD11c^+^ cells [24]. In contrast, the IL-10 levels remained unaffected in B cell or T cell specific IL-10 knockouts on day 10 post infection [24].

Importantly and surprisingly, functional data evaluating the consequences of cell-specific IL-10 deficiency are lacking. It is conceivable that IL-10 is not primarily acting on a systemic level but in a microenvironment where IL-10 secreting cells and the immediate surrounding target cells are spatially and temporarily connected. Therefore, it is of great importance to identify the biologically relevant IL-10 producing cell populations responsible for impairment of LCMV-specific T cell immunity and consequently viral chronicity.

We show here that even though DCs are a major source of IL-10, specific IL-10 deficiency in DCs neither leads to functional improvement of the LCMV-specific T cell response nor does it prevent viral chronicity. Likewise, even though high percentages of NK cells produced IL-10 early after infection, NK cell specific IL-10 knockout mice were not able to clear LCMV and exhibited exhausted T cells. However, depletion of CD4^+^ T cells or monocytes/macrophages resulted in decreased overall amounts of IL-10 mRNA and selective IL-10 deficiency in T cells or myeloid cells prevented viral chronicity, identifying CD4^+^ T cells and monocytes/macrophages as the decisive IL-10 source responsible for suppressing T cell immunity and viral control during high dose LCMV infection.

## Results

### Kinetics of IL-10 production after high dose infection with LCMV Clone 13

**Figure 1:**
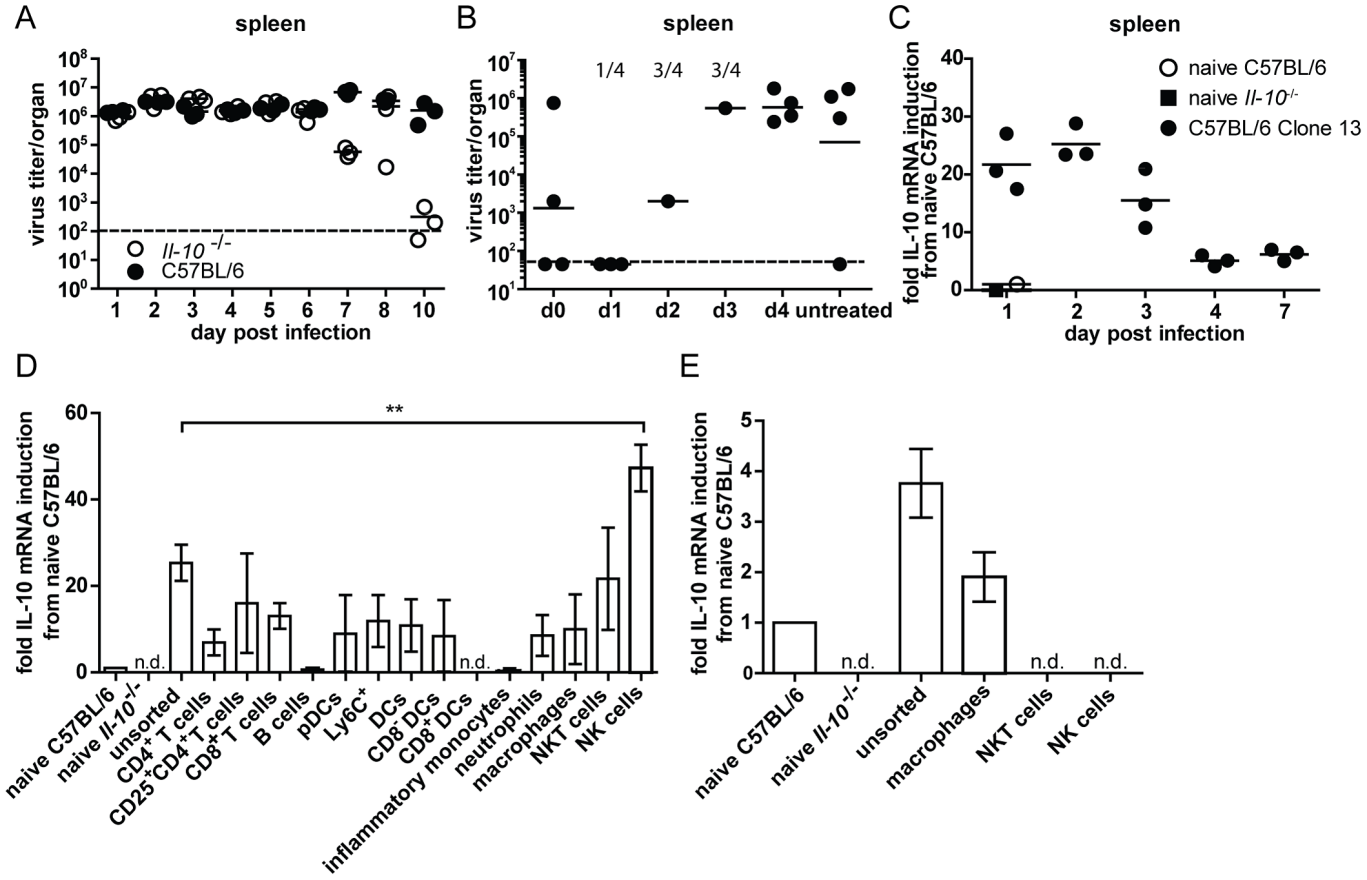
Virus titers in presence and absence of IL-10 and IL-10 mRNA expression levels. (A) C57BL/6 and *Il-10* ^−/−^ mice were infected with LCMV Clone 13 and virus titers were determined in the spleen at the indicated time points. Several time points were reconfirmed in additional experiments. (B) C57BL/6 mice were infected with Clone 13 and treated with αIL-10R at the indicated time point or left untreated. The treatment was repeated in all groups except the untreated group on day 5 post infection. Virus titers in the spleen were analyzed on day 12 post infection. The numbers indicate the number of mice that had to be killed due to immunopathological disease symptoms during the course of the infection. One representative of two experiments is shown. (C) C57BL/6 mice were infected with Clone 13. RNA was extracted from spleen at the indicated time points. cDNA was subjected to IL-10 qPCR. The relative expression was normalized to β-actin. The fold induction relative to the levels in naïve C57BL/6 is depicted. A naïve *Il-10* ^−/−^ mouse served as negative control. Each symbol represents one mouse. One representative of two experiments is shown. (D+E) Splenocytes from C57BL/6 mice were FACS sorted for the indicated cell populations on (D) day 2 and (E) day 5 post infection with LCMV Clone 13 and IL-10 mRNA levels were measured by qPCR. The relative expression was normalized to β-actin. The fold induction relative to the levels in naïve C57BL/6 is shown. Three samples of pooled splenocytes from two mice were analyzed per experiment. A summary of 10 individual experiments with variable repetitions of specific cell types is shown. n.d.: not detectable.

To evaluate the timing when IL-10 determines about persistence or clearance of LCMV, the kinetics of viral titers were compared in C57BL/6 and *Il-10* ^−/−^ mice early after infection (Figure 1A and Figure S1A–E). Interestingly, viral titers were comparable in all tested organs in both mouse strains until day 5/6 post infection. Afterwards, titers gradually decreased in *Il-10* ^−/−^ mice before the virus was cleared around day 10 post infection, whereas the titers remained high in C57BL/6 mice. Next, the question when IL-10R signaling is crucial to establish chronicity was addressed by starting IL-10R blockade at different time points after Clone 13 infection (Figure 1B). Most mice that were treated with αIL-10R antibodies on day 0 and 1 succeeded in clearing LCMV Clone 13, while the groups where αIL-10R treatment was started on day 2 or 3 developed severe immunopathology, which is often observed in situations of concomitant presence of high amount of antigen in presence of high numbers of functional T cells [25], [26]. High viral titers were observed in the group where αIL-10R treatment was started on day 4 post infection, comparable to the untreated group. Similar observations were made in kidney, liver and lung (data not shown). Thus, IL-10 acts during the first two days after infection to prevent immunopathology and to establish viral chronicity.

### Many cell populations are able to produce IL-10 early after high dose LCMV Clone 13 infection

To evaluate the dynamics of IL-10 production within the first days after LCMV infection, IL-10 mRNA levels were measured with daily intervals for the first 4 days. IL-10 mRNA was maximally induced during the first two days after infection and decreased afterwards in spleen (Figure 1C), lung (Figure S1G) and liver (Figure S1I). In the mesenteric lymph node IL-10 mRNA expression peaked on day 3 post infection (Figure S1H). Quantification of IL-10 mRNA expression revealed that the highest amounts of IL-10 mRNA were expressed on day 2 post infection in the spleen (Figure S1F). This prompted us to concentrate further analysis of IL-10 producing cell populations on spleen of day two post infection. FACS sorting of various cell populations and subsequent measurement of IL-10 mRNA levels by qPCR revealed that CD4^+^ T cells, CD8^+^ T cells, CD25^+^ CD4^+^ T cells, pDCs, CD8^−^ DCs, neutrophils, macrophages and NKT cells upregulated IL-10 mRNA compared to naïve C57BL/6 splenocytes (Figure 1D). However, only in purified NK cells the level of IL-10 mRNA increased to levels above those present in unsorted splenocytes. On day 5 post infection, IL-10 mRNA could no longer be detected in NK and NKT cells, whereas expression persisted in macrophages (Figure 1E).

Consistent with these data, analyses in TIGER IL-10-GFP reporter mice revealed around 10% of NK cells expressing GFP, while only between 0.5 and 2% of the NKT cells, DCs, CD4^+^ and CD8^+^ T cells were GFP^+^ on day 2 post infection (Figure S2). On day 5 post infection around 6% of the NK cells and 0.4–2% of the NKT cells, DCs, CD4^+^ and CD8^+^ T cells were GFP^+^.

### Consecutive waves of IL-10 production by different cell populations

**Figure 2:**
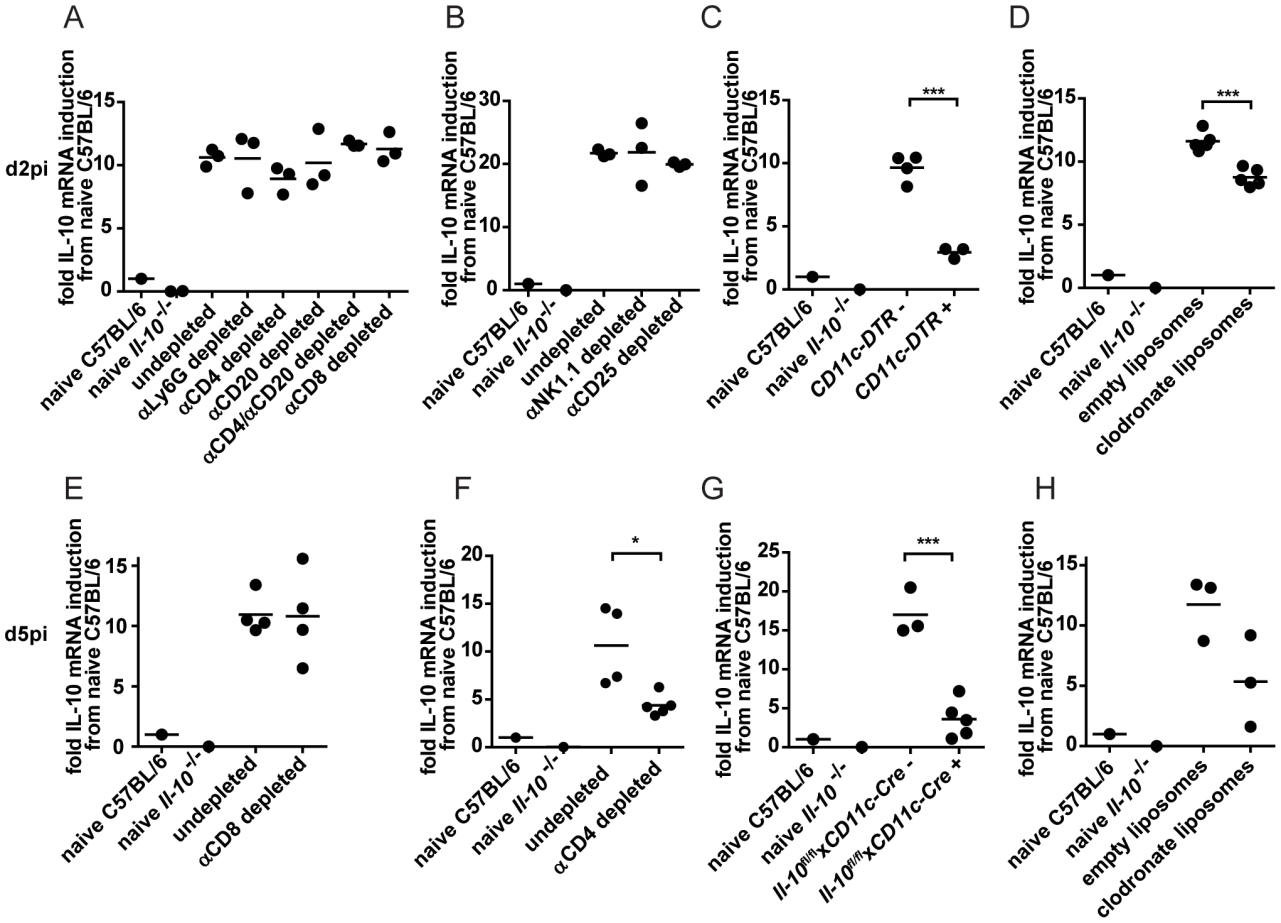
IL-10 mRNA levels on day 2 and 5 post infection after depletion of selective cell populations. (A–D) IL-10 mRNA levels in the spleen were determined on day 2 post LCMV Clone 13 infection by qPCR. (A) C57BL/6 mice were αLy6G, αCD4, αCD20 or αCD8 depleted or left untreated. (B) C57BL/6 mice were αNK1.1 or αCD25 depleted or left untreated. NK depletion: One representative of three experiments depicted. (C) *CD11c-DTR* ^+^ and *CD11c-DTR* ^−^ mice were treated with diphtheria toxin. One representative of two experiments is shown. (D) C57BL/6 mice were treated with clodronate containing liposomes or empty control liposomes. One representative of three experiments is shown. (E–H) IL-10 mRNA levels in the spleen were determined on day 5 post infection with LCMV Clone 13 by qPCR. C57BL/6 mice were (E) αCD8 or (F) αCD4 depleted or left untreated. (G) *Il-10* ^fl/fl^x*CD11c-Cre* ^+^ and *Il-10* ^fl/fl^x*CD11c-Cre* ^−^ mice. (H) C57BL/6 mice were treated with clodronate containing liposomes or empty control liposomes. The relative IL-10 expression was normalized to β-actin. The fold induction relative to the levels in naïve C57BL/6 is shown. A naïve *Il-10* ^−/−^ mouse served as negative control. Each symbol represents one mouse. One representative of two is shown for each experiment.

In order to assess cell populations producing high amounts of IL-10 on a population level instead of on a per cell basis, the percentages of different cell populations of the splenocytes were determined on days 2 and 5 post infection. NK cells constituted 0.96±0.23% of the splenocytes on day 2 and 1.81±0.13% on day 5 post infection while NKT cells accounted for 0.11±0.05% on day 2 and 0.2±0.03% on day 5. B cells were present at 28.7±4.3% on day 2 and at 56.6±2.18% on day 5 while CD4^+^ T cells constituted 7.92±1.47% on day 2 and 8.26±1.35% on day 5 and CD8^+^ T cells accounted for 4.97±1.26% on day 2 and 11.17±0.87% on day 5. DCs represented 0.75±0.15% on day 2 and 0.92±0.18% on day 5 while F4/80^+^ macrophages formed 1.43±0.55% on day 2 and 3.42±0.17% on day 5 (data not shown). To integrate the different abundance of IL-10 producing cell populations, C57BL/6 mice were depleted of specific cell populations and overall IL-10 mRNA levels in splenocytes were compared to undepleted control mice on day 2 post infection. Depletion of neutrophils, CD4^+^ or CD8^+^ T cells, CD25^+^ T cells and B cells did not lead to a decrease in the overall IL-10 mRNA levels (Figure 2A and B). Unexpectedly, depletion of NK-like cells did not result in overall decreased IL-10 mRNA levels (Figure 2B) despite the high IL-10 mRNA expression level in purified cells (Figure 1D). In contrast, depletion of CD11c^+^ cells (DCs, alveolar macrophages and marginal zone macrophages; Figure 2C) led to significantly decreased overall IL-10 mRNA expression. In addition, clodronate liposome meditated depletion of splenic (red pulp, marginal zone and metallophilic [27], but not CD68^+^ [28]) macrophages led to decreased expression of IL-10 mRNA compared to undepleted controls (Figure 2D). Thus, DCs and monocytes/macrophages account for a significant proportion of IL-10 mRNA on day 2 post infection. Analysis on day 5 post infection revealed that CD8^+^ T cell depleted mice expressed similar overall levels of IL-10 mRNA as undepleted controls (Figure 2E), while CD4^+^ T cell depleted, clodronate liposome treated mice and DC specific IL-10 knockout mice (*Il-10* ^fl/fl^x*CD11c-Cre* ^+^ mice) exhibited reduced levels (Figure 2F–H). We chose to use *Il-10* ^fl/fl^x*CD11c-Cre* ^+^ mice instead of DT-treated *CD11c-DTR^+^* mice for the analysis of IL-10 mRNA levels on day 5 post infection due to the described toxicity of DT treatment for 5 days [29] and to avoid secondary consequences on viral control which might be caused due to inefficient T cell priming in absence of DCs [30].

Thus, DCs and monocytes/macrophages account for a first wave of IL-10 production, followed by a second wave of IL-10 production by DCs, CD4^+^ T cells and monocytes/macrophages on day 5 post infection.

### Mast cell, B cell, NK cell and CD11c^+^ cell produced IL-10 is not required to promote T cell exhaustion and viral chronicity

Even though different cell populations expressed IL-10 at different time points after LCMV infection, it is unclear if IL-10 secreted by any particular cell population is promoting viral chronicity and T cell exhaustion. To assess the effects of cell-type specific IL-10 deficiency on T cell effector functions and the resulting decision between chronicity versus clearance of the viral infection, we made use of conditional *IL-10* knockout mice. These mice feature a floxed *IL-10* gene and express the Cre recombinase under control of different cell-type specific promoters. While *Il-10* ^−/−^ mice were able to clear LCMV Clone 13 infection by day 12, *Il-10* ^fl/fl^x*M5-Cre* ^+^ mice, harboring a mast cell specific IL-10 deficiency, were unable to control the infection akin their Cre negative littermate controls and C57BL/6 mice (Figure S3A–D). Single target amplification of the *Il-10* locus of single FACS sorted mast cells from *Il-10* ^fl/fl^x*M5-Cre* ^+^ mice previously confirmed that selectively the deleted version, but never the loxP-flanked product was amplified, while the opposite was true for non-mast cells and Cre-negative control cells [31].

We chose to assess T cell functionality by assessing TNF-α production as a surrogate for polyfunctionality of T cells as it is well established that virtually all TNF-α producing T cells are co-producers of IFN-γ [32], [33] (representative raw data are depicted in Figure S4). We decided to use stimulation with gp33–41 (gp33) as the frequencies of cytokine producing CD8^+^ T cells are higher than after stimulation with np396–404 and therefore not as prone to unspecific variances close to the detection limit (as the percentage of TNF-α producing T cells is fairly low in exhausted T cells; [34]). TNF-α production by antiviral CD4^+^ T cells was measured after stimulation with the viral peptide gp61–80 (p13). In line with the fact that mast cell derived IL-10 was dispensable for the development of a chronic infection, the antiviral CD8^+^ and CD4^+^ T cells of *Il-10* ^fl/fl^x*M5-Cre* ^+^ mice showed an exhausted phenotype in lung and spleen (identified by reduced TNF-α production) unlike *Il-10* ^−/−^ mice (Figure S3E–J). In addition, the number of IFN-γ^+^ CD8^+^ T cells was not significantly different in *Il-10* ^fl/fl^x*M5-Cre* ^+^ and *Il-10* ^fl/fl^x*M5-Cre* ^−^ mice in the spleen (Figure S5A). Thus, mast cell specific IL-10 deficiency neither improves T cell mediated cytokine production nor virus control.

**Figure 3:**
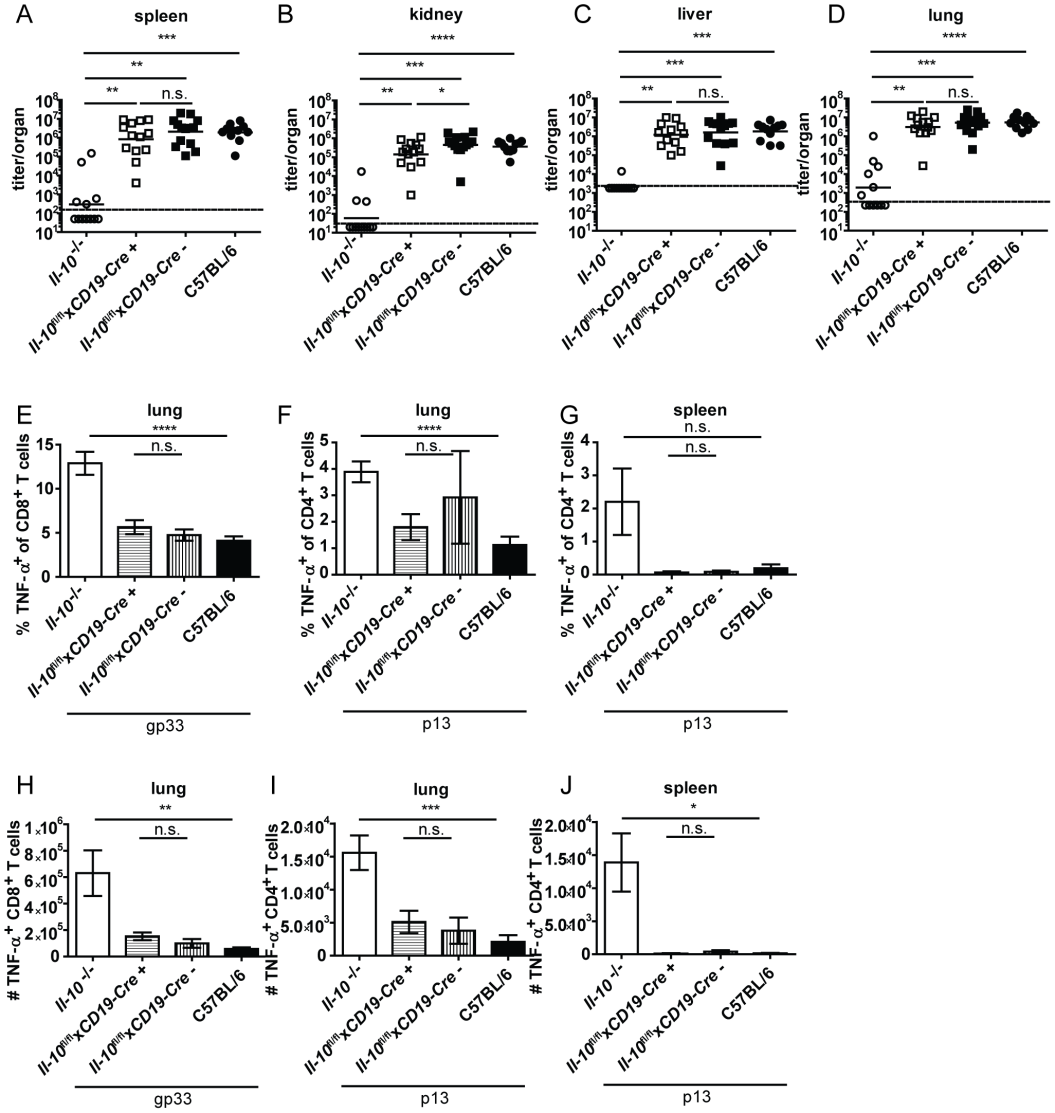
B cell derived IL-10 does not promote viral chronicity and T cell exhaustion. *Il-10* ^−/−^, *Il-10* ^fl/fl^x*CD19-Cre* ^+^, *Il-10* ^fl/fl^x*CD19-Cre* ^−^ and C57BL/6 mice were infected with Clone 13. Virus titers were determined in (A) spleen, (B) kidney, (C) liver and (D) lung on day 10 or day 12 post infection. (A–D) Each symbol represents one individual mouse; the solid lines represent the geometric means, the dashed line the detection limit. Statistical significance was determined with the Mann-Whitney test. (E and H) TNF-α production by CD8^+^ T cells in lung mononuclear cells was determined after 5 hours stimulation with the peptide gp33. TNF-α secretion by CD4^+^ T cells in (F and I) lung mononuclear cells and (G and J) splenocytes was determined after 5 hours stimulation with the peptide p13. Mean and standard deviation of the (E–G) percentages and (H–J) total numbers are shown. A summary of all mice from three independent experiments with 4–5 mice per group is depicted. Statistical significance was determined with unpaired 2-tailed Student's t test. n.s.: not significant; * p<0.05; ** p<0.01; *** p<0.001; *** p<0.0001.

*Il-10* ^fl/fl^x*CD19-Cre* ^+^ mice were previously shown to delete IL-10 specifically in B cells and not or only insignificantly in splenic CD3^+^ T cells, peritoneal macrophages and tail biopsies [35]. Similar to mast cell specific IL-10 knockout mice, B cell specific IL-10 knockout mice displayed comparable virus titers (Figure 3A–D) and TNF-α production by CD4^+^ and CD8^+^ T cells in spleens and lungs to their Cre negative littermates and to C57BL/6 controls (Figure 3E–J; FACS plots of a representative staining are depicted in Figure S4). In addition, the number of IFN-γ^+^ CD8^+^ T cells was similar *Il-10* ^fl/fl^x*CD19-Cre* ^+^ and *Il-10* ^fl/fl^x*CD19-Cre* ^−^ mice in the spleen (Figure S5B; FACS plots of a representative staining are depicted in Figure S4).

**Figure 4:**
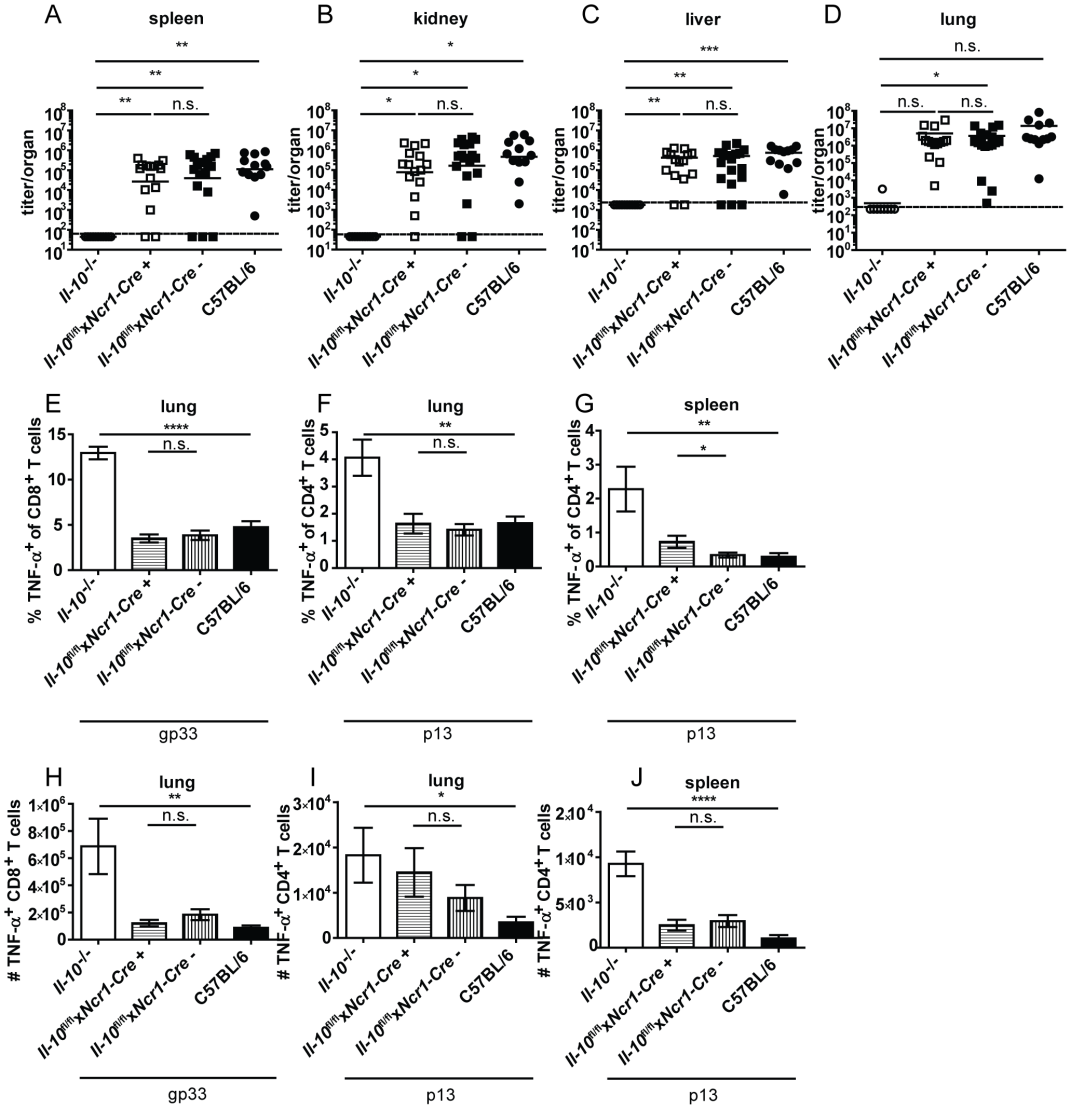
NK cell derived IL-10 does not promote viral chronicity and T cell exhaustion. *Il-10* ^−/−^, *Il-10* ^fl/fl^x*Ncr1-Cre* ^+^, *Il-10* ^fl/fl^x*Ncr1-Cre* ^−^ and C57BL/6 mice were infected with Clone 13. Virus titers were determined in (A) spleen, (B) kidney, (C) liver and (D) lung on day 12 post infection. (A–D) Each symbol represents one individual mouse; the solid lines represent the geometric means, the dashed line the detection limit. Statistical significance was determined with the Mann-Whitney test. (E and H) TNF-α production by CD8^+^ T cells in lung mononuclear cells was determined after 5 hours stimulation with the peptide gp33. TNF-α secretion by CD4^+^ T cells in (F and I) lung mononuclear cells and (G and J) splenocytes was determined after 5 hours stimulation with the peptide p13. Mean and standard deviation of the (E–G) percentages and (H–J) total numbers are shown. A summary of all mice from three independent experiments with 3–10 mice per group is depicted. Statistical significance was determined with unpaired 2-tailed Student's t test. n.s.: not significant; * p<0.05; ** p<0.01; *** p<0.001; *** p<0.0001.

NK cells produced high amounts of IL-10 on a per cell basis on day 2 post infection (Figure 1D). To examine if NK cell derived IL-10 is decisive for Clone 13 infection to become chronic, *Il-10* ^fl/fl^x*Ncr1-Cre* ^+^ mice were derived from *Il-10* ^fl/fl^ and *Ncr1-Cre* ^+^ mice. This mouse strain deleted the *Il-10* gene in 63% of the NK cells (Figure S6A). *Il-10* ^fl/fl^x*Ncr1-Cre* ^+^ mice were infected with Clone 13 and analyzed on day 12 post infection. In agreement with the observation that depletion of NK-like cells did not influence total IL-10 mRNA levels (Figure 2B), *Il-10* ^fl/fl^x*Ncr1-Cre* ^+^ mice displayed high virus titers like their Cre negative littermates and like C57BL/6 mice (Figure 4A–D). In addition, the antiviral CD8^+^ and CD4^+^ T cells showed both low percentages and total numbers of TNF-α^+^ cells and were as exhausted as in the Cre negative littermate controls and as in C57BL/6 mice (Figure 4E–J). Furthermore, the number of IFN-γ^+^ CD8^+^ T cells was similar in *Il-10* ^fl/fl^x*Ncr1-Cre* ^+^ and *Il-10* ^fl/fl^x*Ncr1-Cre* ^−^ mice in the spleen (Figure S5C). Thus, the high amount of IL-10 mRNA on a per cell basis did not translate into a biologically relevant role of NK cell-derived IL-10 for the decision between chronicity and clearance of LCMV infection.

**Figure 5:**
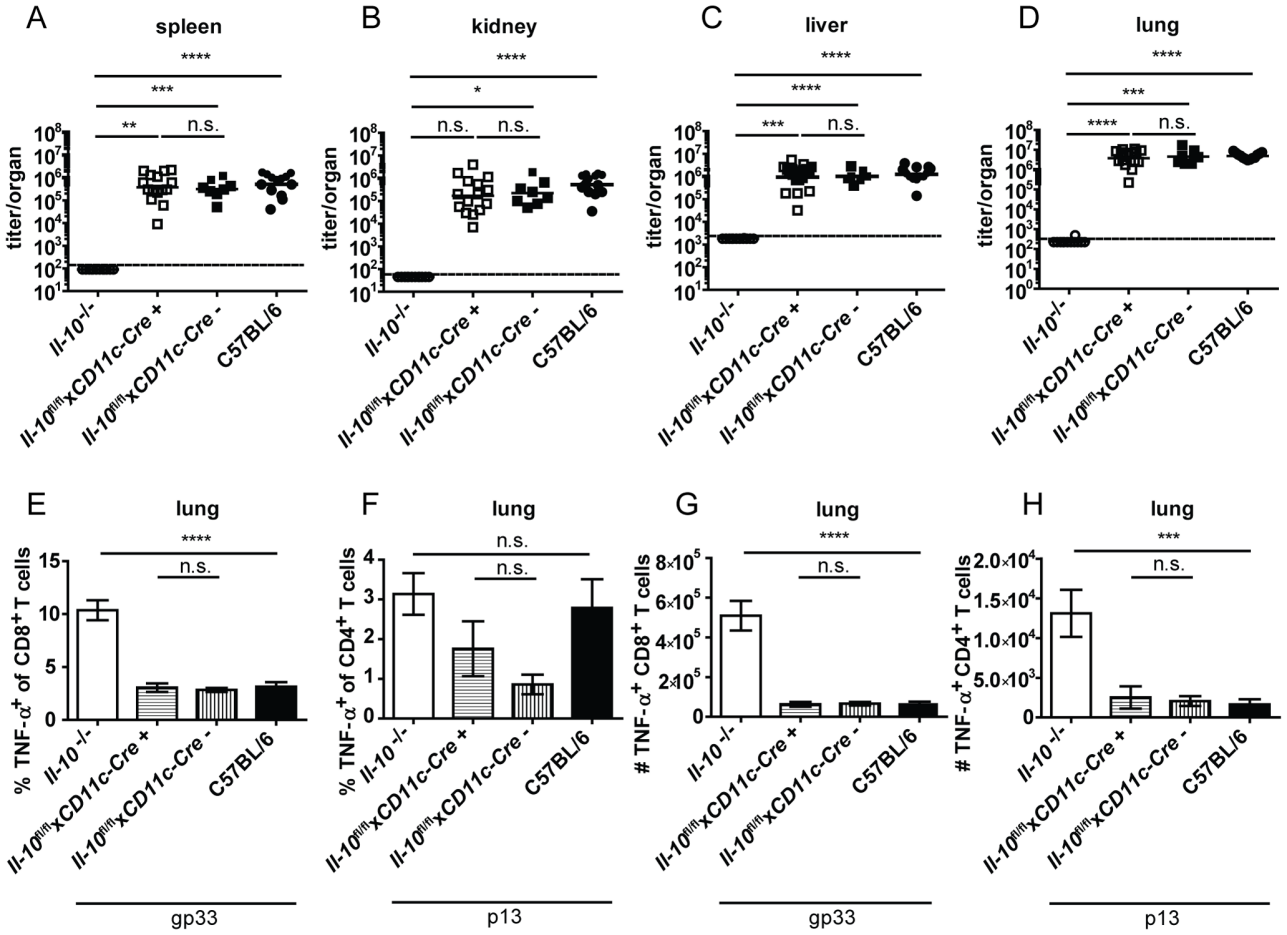
CD11c^+^ cell derived IL-10 does not promote viral chronicity and T cell exhaustion. *Il-10* ^−/−^, *Il-10* ^fl/fl^x*CD11c-Cre* ^+^, *Il-10* ^fl/fl^x*CD11c-Cre* ^−^ and C57BL/6 mice were infected with Clone 13. Virus titers were determined in (A) spleen, (B) kidney, (C) liver and (D) lung on day 10 or 12 post infection. (A–D) Each symbol represents one individual mouse; the solid lines represent the geometric means, the dashed line the detection limit. Statistical significance was determined with the Mann-Whitney test. (E and G) TNF-α production by CD8^+^ T cells in lung mononuclear cells was determined after 5 hours stimulation with the peptide gp33. TNF-α secretion by CD4^+^ T cells in (F and H) lung mononuclear cells was determined after 5 hours stimulation with the peptide p13. Mean and standard deviation of (E and F) the percentages and (G and H) total numbers are shown. A summary of all mice from three independent experiments with 2–8 mice per group is depicted. Statistical significance was determined with unpaired 2-tailed Student's t test. n.s.: not significant; * p<0.05; ** p<0.01; *** p<0.001; *** p<0.0001.

CD8^−^ DCs and pDCs expressed IL-10 mRNA on day 2 post infection (Figure 1D). In accordance with the literature [24], absence of CD11c^+^ cell-derived IL-10 resulted in strongly decreased overall IL-10 mRNA levels on day 2 and 5 post infection in the spleen (Figure 2C and G). *Il-10* ^fl/fl^x*CD11c-Cre* ^+^ mice proved to delete the *Il-10* gene specifically in DCs (Figure S6B). Unexpectedly, LCMV Clone 13 infection was not controlled in *Il-10* ^fl/fl^x*CD11c-Cre* ^+^ mice (Figure 5A–D) and resulted in low percentages and total numbers of TNF-α producing antiviral CD8^+^ and CD4^+^ T cells comparable to littermate and C57BL/6 controls (Figure 5E–H). Furthermore, the number of IFN-γ^+^ CD8^+^ T cells was similar in the spleens of *Il-10* ^fl/fl^x*Ncr1-Cre* ^+^ and *Il-10* ^fl/fl^x*Ncr1-Cre* ^−^ mice (Figure S5D). Thus, even though CD11c^+^ cells contribute substantially to the early overall IL-10 production during LCMV infection (Figure 1D, 2C and G), CD11c^+^ cell derived IL-10 does not seem to play a role for the outcome of the infection and the quality of the antiviral T cell response.

### Monocyte/macrophage derived IL-10 drives chronicity of LCMV infection

**Figure 6:**
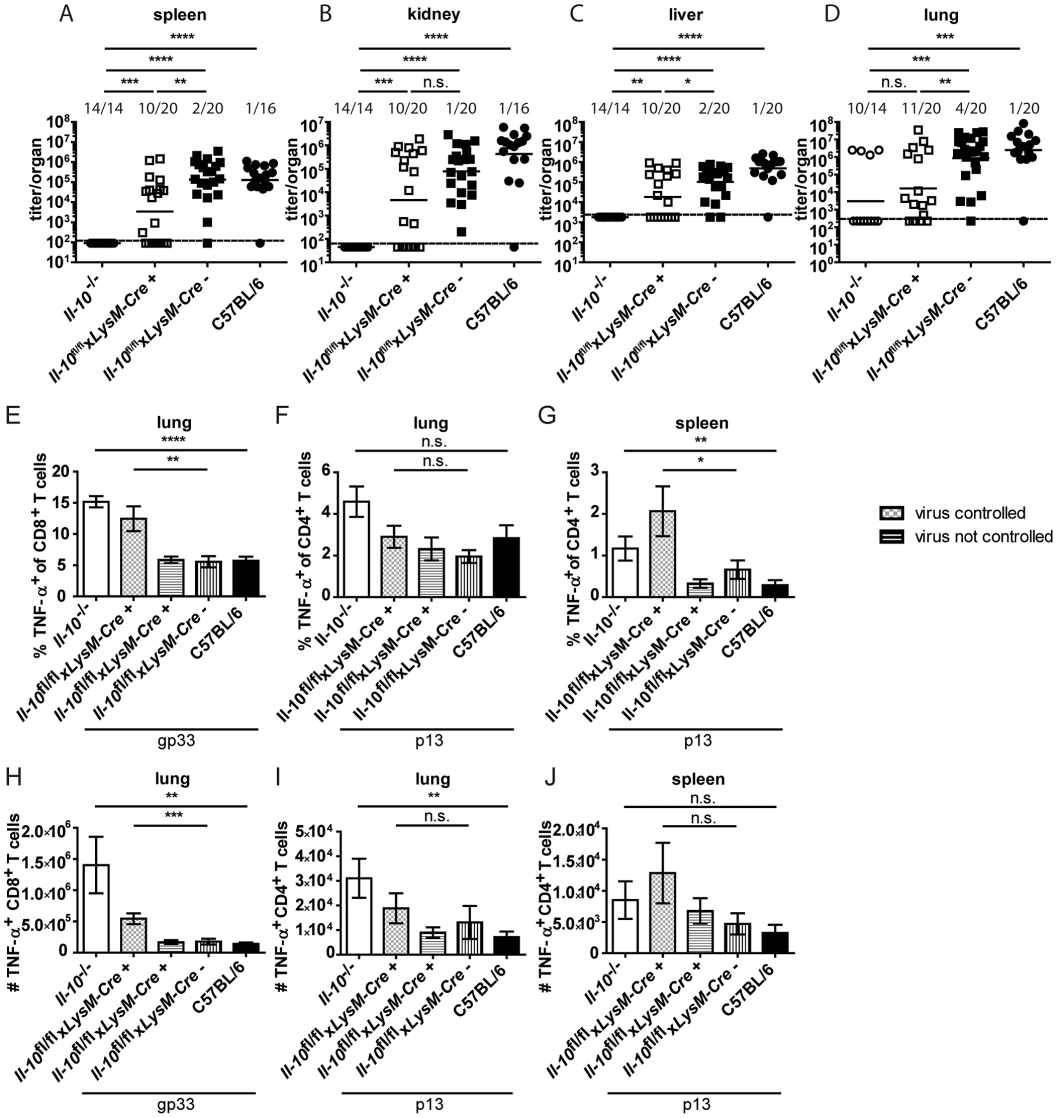
Mononuclear cells are a biologically relevant IL-10 source. *Il-10* ^−/−^, *Il-10* ^fl/fl^x*LysM-Cre* ^+^, *Il-10* ^fl/fl^x*LysM-Cre* ^−^ and C57BL/6 mice were infected with Clone 13. Virus titers were determined in (A) spleen, (B) kidney, (C) liver and (D) lung on day 11 or 12 post infection. Each symbol represents one individual mouse; the solid lines represent the geometric means, the dashed line the detection limit. The numbers indicate how many mice per total mice exhibited virus titers below 10^3^ in spleen and kidney, 5×10^3^ in liver and 10^4^ in lung. Statistical significance was determined with the Mann-Whitney test. (E and H) TNF-α production by CD8^+^ T cells in lung mononuclear cells was determined after 5 hours stimulation with the peptide gp33. TNF-α secretion by CD4^+^ T cells in (F and I) lung mononuclear cells and (G and J) splenocytes was determined after 5 hours stimulation with the peptide p13. Mean and standard deviation of the (E–G) percentages and (H–J) total numbers are shown. Statistical significance was determined with unpaired 2-tailed Student's t test. A summary of all mice from four independent experiments with 2–8 mice per group is depicted. n.s.: not significant; * p<0.05; ** p<0.01; *** p<0.001; *** p<0.0001.

As neutrophils and macrophages produce IL-10 on day 2 and 5 post infection (Figure 1D and E) and as monocyte/macrophage depletion reduced the overall IL-10 mRNA levels (Figure 2D and H), *Il-10* ^fl/fl^x*LysM-Cre* ^+^ mice, in which selectively myeloid cells lack IL-10 production, were infected with Clone 13. *Il-10* ^fl/fl^x*LysM-Cre* ^+^ mice were previously shown to delete IL-10 specifically in neutrophils and macrophages and not or only insignificantly in dendritic cells, T cells and B cells [36]. Interestingly, 50% of the *Il-10* ^fl/fl^x*LysM-Cre* ^+^ mice succeeded in significantly reducing LCMV Clone 13 titers like *Il-10* ^−/−^ mice and unlike the Cre negative littermates and C57BL/6 mice (Figure 6A–D). In line with this, a higher percentage and total number of TNF-α producing T cells was observed in the spleens and lungs of *Il-10* ^fl/fl^x*LysM-Cre* ^+^ mice which could control the infection compared to Cre negative littermate controls, the *Il-10* ^fl/fl^x*LysM-Cre* ^+^ mice which could not control the infection and C57BL/6 mice (Figure 6E–J). In addition, the number of IFN-γ^+^ CD8^+^ T cells was higher in the *Il-10* ^fl/fl^x*LysM-Cre* ^+^ which could reduce the virus load than in *Il-10* ^fl/fl^x*LysM-Cre* ^−^ mice in the spleen (Figure S5E). Thus, myeloid cell derived IL-10 contributes significantly to the biologically relevant effects of IL-10 without accounting for the full phenotype. As depletion of monocytes/macrophages decreased the levels of IL-10 mRNA on days 2 and 5 post infection, while αLy6G depletion failed to do so (Figure 2A), it is likely that monocytes/macrophages are the relevant IL-10 source rather than neutrophils.

### T cell derived IL-10 drives chronicity of the LCMV infection

**Figure 7:**
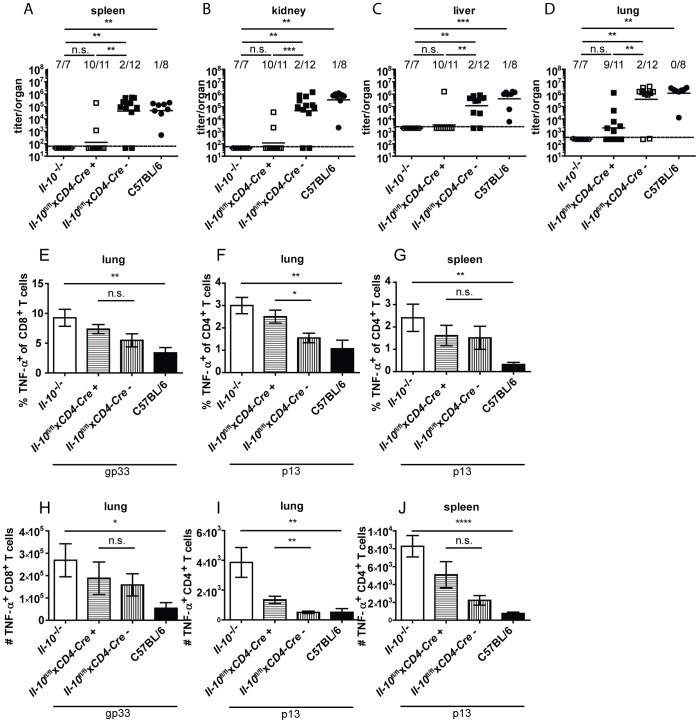
T cells are a biologically relevant IL-10 source. *Il-10* ^−/−^, *Il-10* ^fl/fl^x*CD4-Cre* ^+^, *Il-10* ^fl/fl^x*CD4-Cre* ^−^ and C57BL/6 mice were infected with Clone 13. Virus titers were determined in (A) spleen, (B) kidney, (C) liver and (D) lung on day 10 or 12 post infection. Each symbol represents one individual mouse; the solid lines represent the geometric means, the dashed line the detection limit. The numbers indicate how many mice per total mice exhibited virus titers below 10^3^ in spleen and kidney, 5×10^3^ in liver and 10^4^ in lung. Statistical significance was determined with the Mann-Whitney test. (E and H) TNF-α production by CD8^+^ T cells in lung mononuclear cells was determined after 5 hours stimulation with the peptide gp33. TNF-α secretion by CD4^+^ T cells in (F and I) lung mononuclear cells and (G and J) splenocytes was determined after 5 hours stimulation with the peptide p13. Mean and standard deviation of the (E–G) percentages and (H–J) total numbers are shown. Statistical significance was determined with unpaired 2-tailed Student's t test. A summary of all mice from two independent experiments with 3–8 mice per group is depicted. * p<0.05; ** p<0.01; *** p<0.001; *** p<0.0001.

Based on the finding that CD4^+^ and CD8^+^ T cells produced IL-10 after Clone 13 infection (Figure 1D) and that CD4^+^ T cell depletion decreased IL-10 mRNA levels on day 5 post infection (Figure 2F), we investigated if T cell derived IL-10 complements macrophage derived IL-10 to exert it's biological effects. *Il-10* ^fl/fl^x*CD4-Cre* ^+^ mice were shown previously to delete the *Il-10* gene specifically in T cells [37]. Interestingly, almost all *Il-10* ^fl/fl^x*CD4-Cre* ^+^ mice succeeded in clearing Clone 13 within 12 days comparable to *Il-10* ^−/−^ mice and opposed to Cre negative littermate controls and C57BL/6 mice (Figure 7A–D). In line with these data, frequencies and numbers of TNF-α producing T cells were increased in mice with selective IL-10 deficiency in T cells compared to Cre negative littermate controls and C57BL/6 mice (Figure 7E–J). Likewise, the number of IFN-γ^+^ CD8^+^ T cells was higher in the spleens of *Il-10* ^fl/fl^x*CD4-Cre* ^+^ than in *Il-10* ^fl/fl^x*CD4-Cre* ^−^ mice (Figure S5F). However, the percentage and total number of TNF-α producing antiviral CD4^+^ and CD8^+^ T cells was lower in *Il-10* ^fl/fl^x*CD4-Cre* ^+^ mice compared to *Il-10* ^−/−^ mice, underlining the fact that macrophages and potentially also other cell populations contribute to the overall relevant IL-10 production. It should be noted that a slight increase in the viral inoculum led to increased incidence of viral chronicity in *Il-10* ^fl/fl^x*CD4-Cre* ^+^ mice while most *Il-10* ^−/−^ mice were still able to clear the infection (data not shown), suggesting that IL-10 derived from T cells and myeloid cells act together to exert its full biological activity.

To further assess which CD4^+^ T cell subpopulation might be mainly responsible for the IL-10 production, TIGER IL-10 reporter mice were analyzed for GFP expression on day 10 post infection in T_FH_ and T_H_1 cells using PSGL1 and Ly6C as surrogate markers (with PSGL1^+^ Ly6C^+^ CD4^+^ T cells representing T_H_1 cells and PSGL1^−^ Ly6C^−^ cells representing T_FH_ cells; Figure S7A–F). GFP^+^ cells could be detected in both subpopulations. Likewise, GFP expression could be detected in a subpopulation of Neuropilin^+^ natural T_regs._ However, the biggest increase compared to the naïve situation could be detected in Neuropilin^−^ induced T_regs_ (Figure S7G–I). The finding that induced T_regs_ as opposed to T_H_1 cells are probably responsible for the majority of the CD4^+^ T cell derived IL-10 is supported by the finding that only very few IFN-γ^+^ CD4^+^ T cells co-produced IL-10 (Figure S7J–K).

## Discussion

Several cell populations are capable of producing IL-10 [10], [38]. DCs, B cells, macrophages, CD4^+^ and CD8^+^ T cells were previously shown to produce IL-10 after LCMV infection [2], [3], [22], [24]. However, data on functional consequences of IL-10 deficiency in defined cell types were lacking so far. In agreement with previous studies [2], [3], [22], [24] we demonstrate here that DCs, B cells, macrophages, CD4^+^ and CD8^+^ T cells, but also neutrophils, NK cells and NKT cells produce IL-10 in the early phase of high dose LCMV Clone 13 infection. These diverse IL-10 producing cell types potentially all play important and distinct roles in suppressing the host's immune response. However, we clearly show that myeloid and T cell derived IL-10 is critical in promoting viral chronicity and T cell exhaustion, while IL-10 derived from other sources, including DCs, only contributes marginally - if at all - to the suppression of the antiviral immune response. Since depletion of monocytes/macrophages but not neutrophils led to markedly reduced IL-10 mRNA levels, we conclude that monocyte/macrophage derived IL-10 accounts for the reduced virus titer and increased cytokine production by antiviral T cells occurring in the myeloid cell specific IL-10 knockout mice.

IL-10 drives viral persistence in a number of chronic infections. Elevated IL-10 levels were found to correlate with HIV replication in humans and chronicity during HCV and HBV infection [4], [5], [6]. Likewise, disruption of IL-10 receptor signaling enhances CD8^+^ effector functions of T cells isolated from HIV, HCV and HBV infected individuals [6], [7], [8], [9] like after LCMV infection [2], [3].

The dominant IL-10 producing cell population varies with different infections, likely reflecting differences in pathogen-specific responses and tissue-specific immune regulation. Usually, IL-10 is produced by multiple cell populations [10]. During HIV infection, IL-10 production by T cells, B cells, NK cells and monocytes was reported [4] with monocytes/macrophages being the subset producing highest levels of IL-10 in the peripheral blood [39], [40], [41]. In contrast, B cell derived IL-10 is important during HBV infection [6]. During murine cytomegalovirus (MCMV) infection, IL-10 can be produced by CD4^+^ T cells, B cells, inflammatory macrophages, dendritic cells and NK cells [19], [35], [42], [43]. The biologically relevant in vivo sources of IL-10 in MCMV infection were identified as CD11c^+^ cells and to some extent macrophages. IL-10 from these cellular sources led to the suppression of NK/DC cross-talk and consequently to curtailed CD4^+^ T cell priming, thereby promoting prolonged lytic MCMV [19]. In addition, B cell produced IL-10 was shown to suppress the CD8^+^ T cell response in the spleen during MCMV infection [35]. During *Leishmania major* infection anti-IL-10R treatment leads to sterile cure of infection with complete elimination of the parasite. In this infection model, CD4^+^ and CD8^+^ T cell derived IL-10 plays an essential role for the establishment of pathogen persistence [44]. Thus, the different importance of certain cell populations serving as IL-10 source might depend on the specific tissue, the cell population affected and the time after infection.

It was recently shown that myeloid suppressor cells inhibit CD8^+^ T cell proliferation during chronic LCMV infection [45]. However, the fact that *in vitro* co-cultivated myeloid suppressor cells could still inhibit CD8^+^ T cell proliferation in presence of an anti-IL-10R antibody [45] renders it rather unlikely that myeloid suppressor cells are a relevant source of IL-10, assuming that IL-10 acts directly on the CD8^+^ T cells.

CD4^+^ T cell depletion led to a decrease in the overall IL-10 mRNA levels on day 5 post infection with LCMV while depletion of CD8^+^ T cells had no effect (Figure 2). Therefore, it is likely that CD4^+^ T cell derived IL-10 is more important than CD8^+^ T cell derived IL-10. IL-10 was initially identified as T_H_2 cytokine [46], but can also be produced by T_H_1, T_H_17, T_FH_, natural and induced T_regs_ [47]. As αCD25 depletion did not decrease IL-10 mRNA levels on day 2 post infection, natural T_regs_ do not seem to be relevant IL-10 producers during LCMV infection. These data were supported by the finding that IL-10^+^ Neuropilin^+^ natural T_regs_ did not accumulate upon infection. In contrast to natural T_regs_, IL-10-producing Neuropilin^−^ induced T_regs_ accumulated by day 10 after LCMV infection and likely constitute a subpopulation of CD4^+^ T cells that is responsible to promote chronicity of the infection, at least on day 10 post infection when LCMV chronicity is already established. Accumulation of induced T_regs_ during established chronic LCMV infection is in agreement with previous studies [48], [49].

Interestingly, the depletion of monocytes/macrophages decreased IL-10 mRNA levels on days 2 and 5 post infection while CD4^+^ T cell depletion led to reduced concentrations of IL-10 mRNA only on day 5. This implies a cooperation of macrophages and CD4^+^ T cells in a way that the macrophages provide a first wave of IL-10 until, in a second wave, different subtypes of CD4^+^ T cells (T_H_1, T_FH_, natural T_regs_ and induced T_regs_ with induced T_regs_ exhibiting the highest percentage of IL-10 producing cells) start to significantly contribute to the overall IL-10 production. Together with the fact that all analyzed CD4^+^ T cell subpopulations contribute to IL-10 production, it seems as if multiple cell types share the task of IL-10 secretion in a time-dependent manner. It is possible that IL-10 production by monocytes/macrophages is preceded by a first wave of IL-10 production by NK cells. However, absence of NK cell derived IL-10 did not promote control of high dose LCMV infection, suggesting that IL-10 production by monocytes/macrophages plays a more dominant role. It should be noted, however, that the NK cell-specific *Il-10* deletion efficacy was only 63% (Figure S6 and [50]), but in combination with the observation that NK cell depletion did not lead to an overall reduction in IL-10 mRNA on day 2 post infection it is rather unlikely that NK cell produced IL-10 plays a decisive role in promoting LCMV chronicity.

The observation that dendritic cells produce IL-10 and that the IL-10 mRNA levels decreased dramatically in absence of CD11c^+^ cells (Figure 2C) is consistent with the finding that *Il-10* ^fl/fl^x*CD11c-Cre* mice expressed considerably less IL-10 (Figure 2G and [24]). Importantly and to some extend surprisingly, we extend this observation by showing that IL-10 produced by CD11c^+^ cells lacks functional consequences with respect to antiviral T cell function or the establishment of viral chronicity (Figure 5).

It is likely that the precise anatomical localization and kinetics of the IL-10 secreting cells in relation to the responding cell is of considerable importance. This would be one possible explanation for the observation that IL-10 produced by CD11c^+^ cells does not influence the outcome of the infection even though IL-10 promoter activity could be detected in DCs of IL-10 reporter mice and even though depletion of CD11c^+^ cells dramatically decreased the overall amount of IL-10 mRNA. On the other hand, post-transcriptional regulation of IL-10 may vary in different cell types, such that quantitative differences evidenced by mRNA analyses might not translate to the protein level [51], [52].

One potential caveat of IL-10 mRNA analysis after *in vivo* depletion of certain cell populations for several days into LCMV infection is that the absence of specific cell types may alter virus titers which might in turn affect the IL-10 production by other cells types. In addition, depletion of CD11c^+^ cells by DT administration in *CD11c-DTR* ^+^ mice was shown to cause neutrophilia [53]. So, if neutrophils contributed to IL-10 production significantly, their increased abundance might have masked the decrease in IL-10 mRNA levels in diphtheria toxin treated *CD11c-DTR* mice.

Our data do not permit a conclusion on whether the more potent antiviral T cell responses observed in absence of T cell or myeloid-derived IL-10 are a cause or a consequence of the concomitantly reduced virus titers, but they underscore an important role of IL-10 in shaping the mutual relationship between prolonged high antigen load and decreased T cell function [26]. It has to be noted that only a defined LCMV inoculum is controlled in the T specific IL-10 knockout mice, while a wider range of inocula is controlled in full IL-10 deficient mice. This is likely one reason why the full IL-10 knockout mice show more robust T cell responses compared to the cell-specific knockout mice, yet these small increases might be sufficient under these experimental settings to allow for virus control.

At present it is unclear on which cell types the T cell or myeloid cell derived IL-10 acts to promote viral chronicity but it is likely that multiple immune cell subsets will be targeted. It was recently shown that splenic DCs treated *in vitro* with type I IFN produce IL-10 [24] and that anti-IFNR1 blockade led to reduced numbers of IL-10^+^ PD-L1^+^ immunoregulatory DCs which in turn stimulated elevated numbers of IFN-γ^+^ CD4^+^ T cells and a decrease in viral titers [54]. It is likely that type I IFN also directly induces IL-10 production in macrophages at the early time points after infection. However, as the amounts of IFN decline at later time points [55], this might indicate that CD4^+^ T cell derived IL-10 is induced by other mechanisms.

In this context we have measured type I IFNs in the serum of C57BL/6 and *Il-10* ^−/−^ mice and we have observed increased concentrations of type I IFN in *Il-10* ^−/−^ mice on day 3 post infection (data not shown). As Type I IFNs are an essential signal 3 in LCMV to drive T cell expansion [56], [57] and effector cell differentiation [58], it is possible that increased peak levels of type I IFNs might contribute to more effective T cell responses which in turn would accelerate virus control. It is also possible that increased levels of type I IFNs in *Il-10* ^−/−^ mice might have a direct effect on LCMV control, but this is rather unlikely as we do not see any changes in viral load in the first 5 days post infection.

As we determined that IL-10 has to act within the first 2 days it is very likely that initially innate immune cells or non-hematopoietic cells are targeted by IL-10. Indeed, we have preliminary data that IL-10 does not directly act on CD4^+^ or CD8^+^ T cells and on DCs (data nor shown). The innate target cells could be macrophages themselves (being infected by LCMV) which are modulated by IL-10 such that they would better sustain viral replication, potentially by inhibition of NO production as it was shown for parasite infection [59] or by inhibition of TNF-α and IFN-γ production [60]. We believe that a direct effect of IL-10 on virus production is rather unlikely as we have seen in our *in vivo* kinetics of viral loads that differences between C57BL/6 and *Il-10* ^−/−^ mice only become apparent after day 6 of infection (Fig. 1).

Alternatively, NK cells might be possible IL-10 target cells as they are mainly situated in the red pulp of the spleen where they might be targeted by macrophage-secreted IL-10. In MCMV infection it has been shown that NK cells are over-activated in absence of IL-10 and are undergoing increased levels of activation-induced cell death [43]. As it has been shown in the LCMV system that activated NK cells can reduce the number of activated virus-specific CD4^+^ T cells [61], it is conceivable that potentially reduced numbers of NK cells in the absence of IL-10 would translate into better preserved virus-specific CD4^+^ (and CD8^+^) T cell responses.

With respect to the secondary requirement of CD4^+^ T cell derived IL-10 driving viral chronicity we hypothesize that by day 4–5 post infection, activated CD4^+^ T cells will not only be in secondary lymphoid organs but also in (infected) peripheral tissues where their IL-10 secretion might affect local immune control. Based on our observation that IL-10 does not directly act on T cells and based on additional observations that the per cell killing efficiency of CD8^+^ T cells (when using optimally peptide-loaded APCs) is not altered in presence or absence of IL-10 (data not shown), we postulate that either the increased frequencies of T cells in peripheral tissues lead to better virus control or that in addition presence of (CD4^+^ T cell secreted) IL-10 would impact on the antigen presentation on infected cells. IL-10 is known to down-modulate MHC expression and it could thereby reduce sensitivity towards LCMV infected cells.

In summary, our data clearly indicate a non-redundant role of T cell and myeloid cell derived IL-10 in facilitating viral chronicity and T cell exhaustion after LCMV Clone 13 infection as opposed to DCs, which were so far proclaimed to exert this function.

## Materials and Methods

### Virus and viral peptides

LCMV Clone 13 was propagated and titrated as described [62]. The viral peptides gp33–41 (gp33; KAVYNFATM), np396–404 (np396; FQPQNGQFI) and gp61–80 (p13; LNGPDIYKGVYQFKSVEFD) were purchased from NeoMPS (Strasbourg, France).

### Mice

Animal experiments were performed according to the guidelines of the animal experimentation law (SR 455.163; TVV) of the Swiss Federal Government. The protocol was approved by the Cantonal Veterinary Office (animal experimentation number 146/2008 and 127/2011).

*Ncr1-Cre* mice [50] were crossed to *Il-10* ^fl/fl^ mice [37] yielding *Il-10* ^fl/fl^x*Ncr1-Cre* mice.

C57BL/6 (Janvier Elevage, Le Genest Staint Isle, France), *Il-10* ^fl/fl^x*CD4-Cre* [37], *Il-10* ^fl/fl^x*LysM-Cre* [36], *Il-10* ^fl/fl^x*CD11c-Cre* [24], *Il-10* ^fl/*fl*^ *xCD19-Cre* [35], *Il-10* ^fl/fl^x*M5-Cre* [31], *Il-10* ^−/−^ [63], TIGER [64] and *CD11c-DTR/GFP* [29] mice were kept under specific pathogen-free conditions and were intravenously (i.v.) infected with LCMV Clone 13. Each virus stock was titrated *in vivo* and injected at doses that led to a chronic infection in C57BL/6 mice and to reduced virus titers by day 10 to 12 in most of the *Il-10* ^−/−^ mice (between 3×10^5^ and 10^6^ ffu per mouse).

### Antibodies and *in vivo* depletion of cell populations

Antibodies used for flow cytometry were purchased from BD Pharmingen (Allschwil, Switzerland), Biolegend (LucernaChem, Luzern, Switzerland) and R&D Systems (Abingdon, England). For the depletion of neutrophils, mice were i.p. injected with 500 µg αLy6G (clone 1A8, BioXCell, West Lebanon, NH, USA) on day -2 and one hour before infection (99.8% reduction in the percentage of Ly6G^+^ cells). For the depletion of CD4^+^ T cells, mice were i.p. injected with 200 µg αCD4 (YTS 191.1) on day -2 and one hour before infection (depletion efficacy of 97% while the percentage of CD8^+^ T cells increased slightly). For the depletion of B cells, mice were i.p. injected with 250 µg αCD20 (18B12; IgG2a; [65]) on day -2 and one hour before infection (depletion efficacy of 98% while the percentage of CD8^+^ T cells and CD4^+^ T cells increased compared to mice treated with an isotpye control). For the depletion of CD8^+^ T cells, mice were i.p. injected with 200 µg αCD8 (YTS 169.4) on day -2 and one hour before infection (depletion efficacy of 99% while the CD4^+^ T cell population stayed unaffected). Depletion of NK-like cells was performed by i.p. administration of 300 µg αNK1.1 (PK136, BioXCell) on days -3 and -1 (depletion efficacy of 96% while the percentage of CD3^+^ cells was not affected). For the depletion of regulatory CD4^+^ T cells mice were i.p. injected with 200 µg of αCD25 (PC-61.5.3, BioXCell) 3 and 1 day before infection (depletion efficacy of 70%). For depletion of CD11c^+^ cells, *CD11c-DTR* ^+^ and *CD11c-DTR* ^−^ mice were i.p. treated with 200 ng diphtheria toxin (Sigma-Aldrich, Buchs, Switzerland) on days -2, -1 and 0 (depletion efficacy of 95% while the percentage of B cells was not affected). For depletion of monocytes/macrophages mice were i.p. treated with liposomes containing 1 mg clodronate [27] or equal amounts of empty control liposomes on days -7, -5, -2 and 0 (depletion efficacy of 96% while the percentage of B cells and T cells was not affected). The depleted mice were analyzed on day 2 post infection. When analyzed on day 5 post infection, the depleting agent was continuously administered every second to third day.

### Isolation and stimulation of lymphocytes and flow cytometry

Splenocytes and mononuclear cells from lungs were prepared, stimulated and stained as previously described [26]. Phorbol 12-myristate 13-acetate (PMA) and Ionomycin were purchased from Sigma and used at 50 ng/ml and 500 ng/ml, respectively. Multiparameter flow cytometric analysis was performed using a FACS LSRII flow cytometer (BD Biosciences, Allschwil, Switzerland) with FACSDiva software. Analysis was performed using FlowJo software (Tree Star, San Carlos, CA). Sorting was performed using a FACS Aria flow cytometer (BD Biosciences, Allschwil, Switzerland). The sorted cell populations were defined as follows: CD4^+^ T cells: CD4^+^ CD25^−^; CD25^+^ CD4^+^ T cells: CD4^+^ CD25^+^; CD8^+^ T cells: CD8^+^ CD4^−^; B cells: CD4^−^ CD19^+^; pDCs: B220^+^ PDCA-1^+^ Ly6C^+^; DCs: CD11c^+^ I-A/I-E^+^; CD8^−^ DCs: CD11c^+^ I-A/I-E^+^ CD8^−^; CD8^+^ DCs: CD11c^+^ I-A/I-E^+^ CD8^+^; inflammatory monocytes: CD11b^+^ Ly6C^+^ Ly6G^−^; neutrophils: CD11b^+^ Ly6C^−^ Ly6G^+^; macrophages: CD11b^int^ F4/80^+^; NKT cells: CD3^+^ NK1.1^+^; NK cells: CD3^−^ NK1.1^+^.

### Enrichment of NK cells and DCs and Southern blot

NK cells were isolated from splenocytes on day 7 after i.p. infection with 5×10^4^ pfu MCMV Smith by magnetic negative selection of CD3^+^ and CD19^+^ cells and subsequent FACS sorting of CD3^−^ NK1.1^+^ cells. DCs were isolated from splenocytes on day 7 after s.c. injection with 10^6^ B16-Flt3L cells by magnetic negative selection of CD3^+^ and CD19^+^ cells and subsequent FACS sorting of CD11c^+^ MHCII^+^ cells. Southern Blot was performed as described [37].

### RNA isolation, reverse transcription and IL-10 qPCR

Total RNA was isolated with Trizol (Invitrogen, Basel, Switzerland) according to the manufacturer's instructions. For isolation of RNA from whole organs, the organ was homogenized with a stainless steel bead for 1.5 min at 25 Hz in a Qiagen Tissue Lyser. 1 µg of RNA was reverse transcribed with M-MLV Reverse Transcriptase RNase, H Minus (Promega, Dübendorf, Switzerland). Real-time PCR was performed using a Rotorgene 3000 instrument (Corbett Research, Eight Miles Plains, Australia) to measure SYBR green (Sensi Mix, R&D Systems) incorporation. The following primer sets were used: IL-10: 5′-ATGCTGCCTGCTCTTACTGACTG-3′ and 5′-CCCAAGTAACCCTTAAATCCTGC-3′ [66]; β-actin: 5′-CCCTGAAGTACCCCATTGAAC-3′ and 5′-CTTTTCACGGTTGGCCTTAG-3′. The amount of IL-10 mRNA was normalized to β-actin RNA levels for each sample and was expressed relative to the levels of uninfected control mice.

### Statistical analysis

The two-tailed unpaired *t* test was applied for statistical analysis when the distribution was Gaussian. Otherwise, the Mann-Whitney test was performed. * p<0.05; ** p<0.01; ***p<0.001; **** p<0.0001; n.s. not significant.

## Supporting Information

Figure S1 Kinetics of virus titers in presence and absence of IL-10 and IL-10 mRNA expression levels early after LCMV Clone 13 infection.
(TIF)

Figure S2 IL-10-GFP reporter expression on days 2 and 5 post infection.
(TIF)

Figure S3 Mast cell derived IL-10 does not promote viral chronicity and T cell exhaustion.
(TIF)

Figure S4 B cell derived IL-10 does not promote viral chronicity and T cell exhaustion.
(TIF)

Figure S5 IFN-γ production in absence of mast cell-, B cell-, NK cell-, DC-, myeloid cell- and T cell-derived IL-10.
(TIF)

Figure S6 Specific deletion of the *Il-10* gene in NK cells of *Il-10* ^fl/fl^xNcr1-Cre and in DCs of *Il-10* ^fl/fl^xCD11c-Cre mice.
(TIF)

Figure S7 IL-10 production by different CD4^+^ T cell subpopulations.
(TIF)
