# Supplementary figures and images for: Macrophage and T Cell Produced IL-10 Promotes Viral Chronicity
Source: PLoS Pathog. 2013 Nov 7;9(11):e1003735. doi: 10.1371/journal.ppat.1003735 (PMC3820745; doi:10.1371/journal.ppat.1003735)

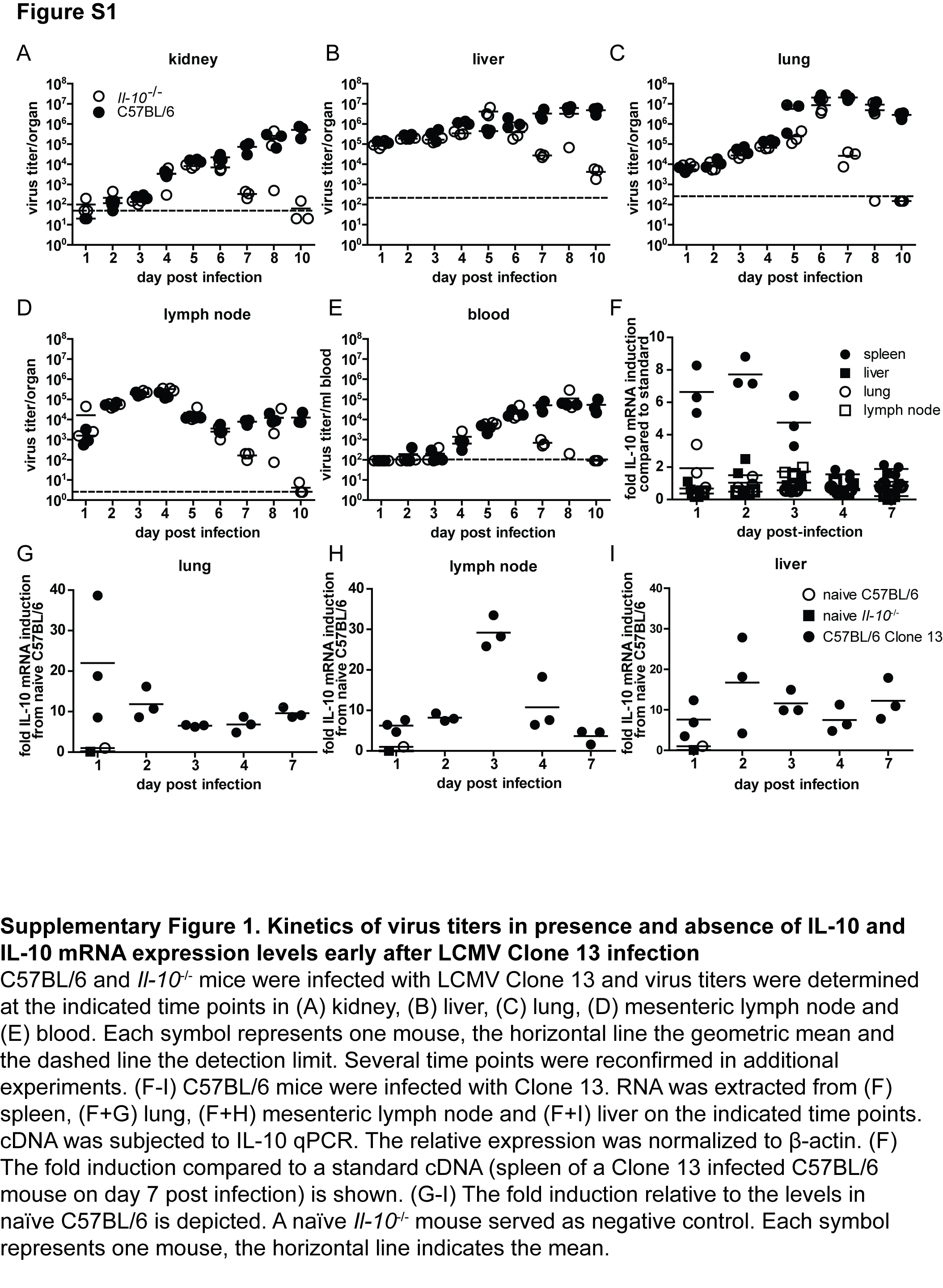

Supplement: Figure S1 — Kinetics of virus titers in presence and absence of IL-10 and IL-10 mRNA expression levels early after LCMV Clone 13 infection. (TIF) [file ppat.1003735.s001.tif]

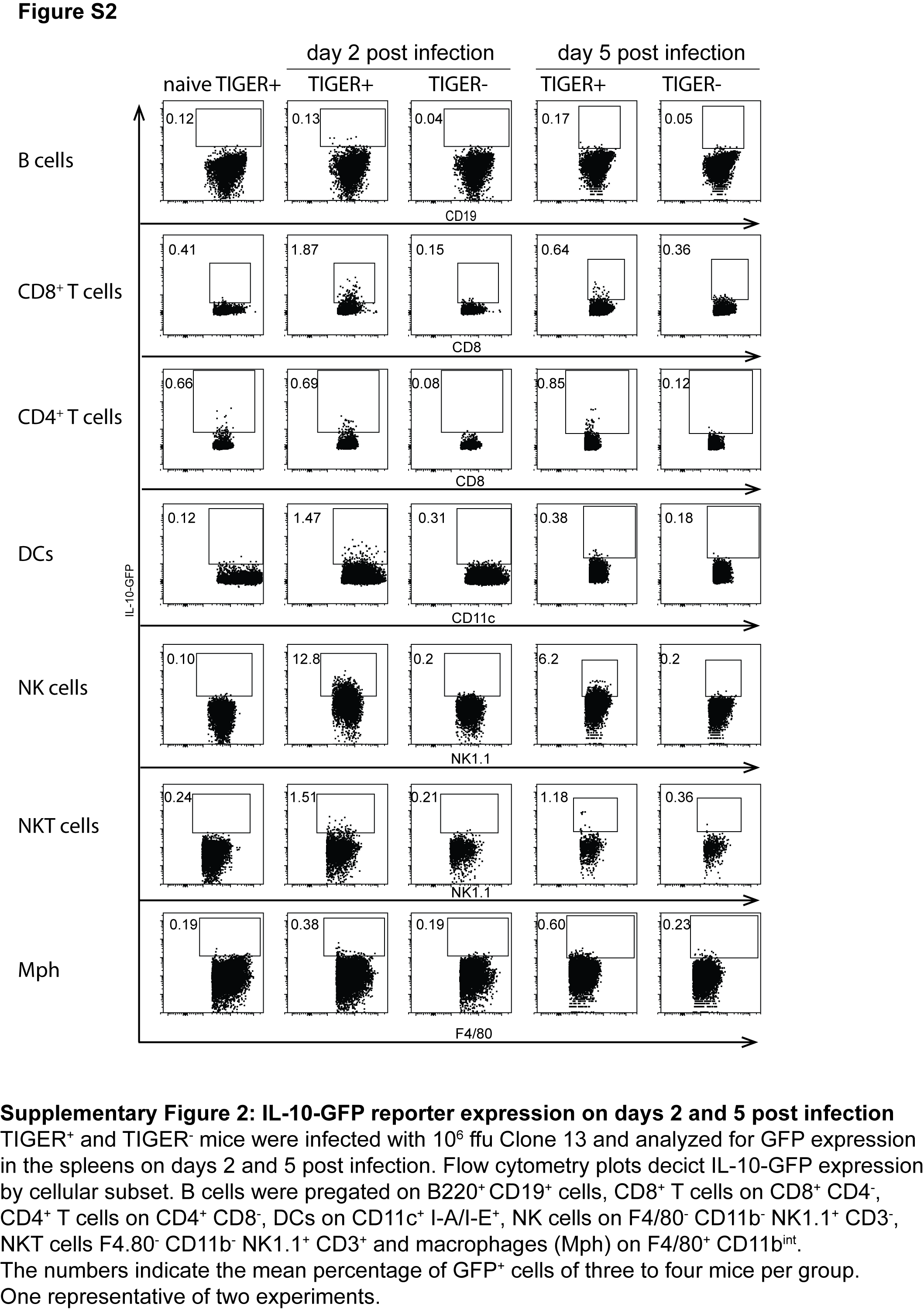

Supplement: Figure S2 — IL-10-GFP reporter expression on days 2 and 5 post infection. (TIF) [file ppat.1003735.s002.tif]

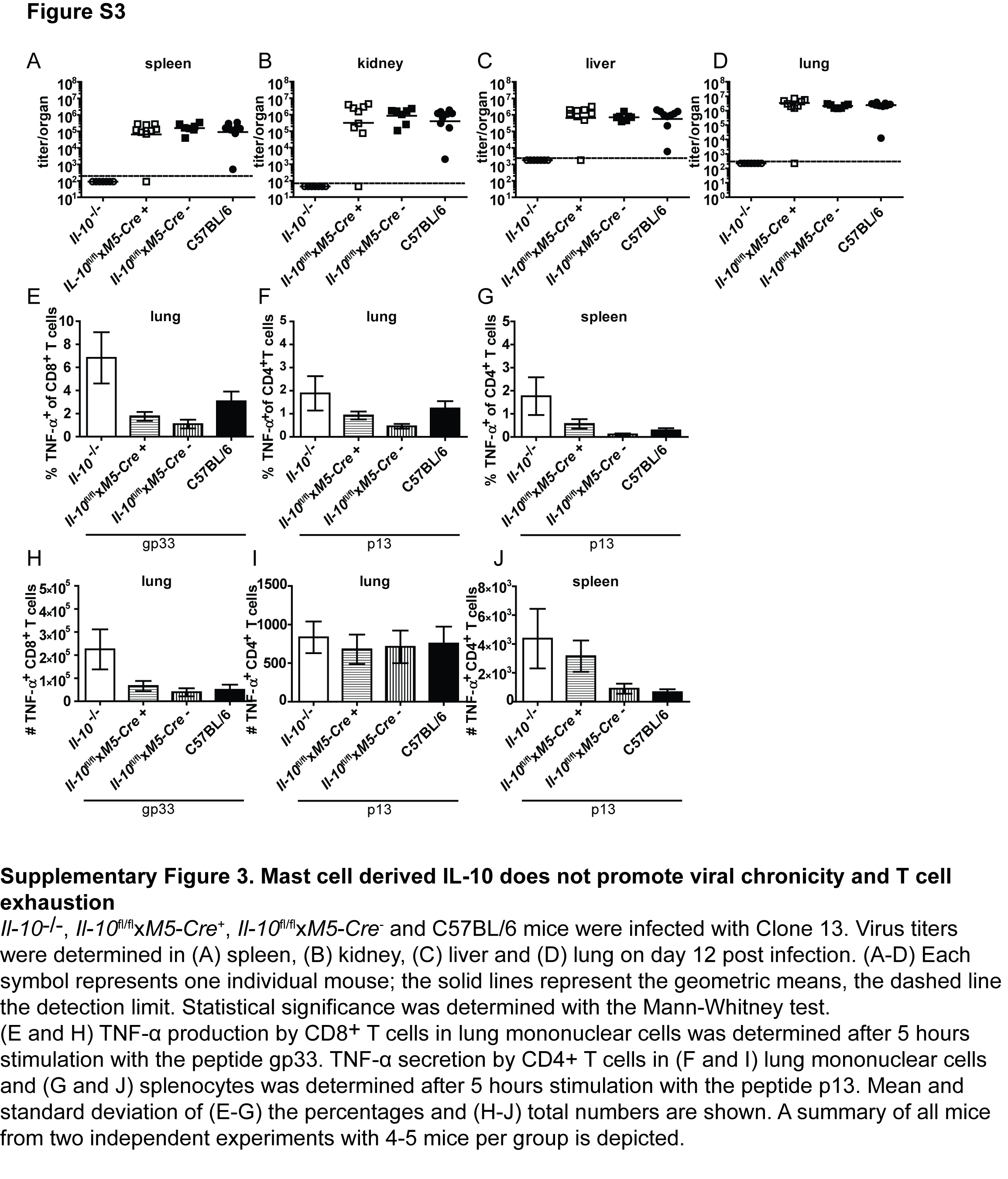

Supplement: Figure S3 — Mast cell derived IL-10 does not promote viral chronicity and T cell exhaustion. (TIF) [file ppat.1003735.s003.tif]

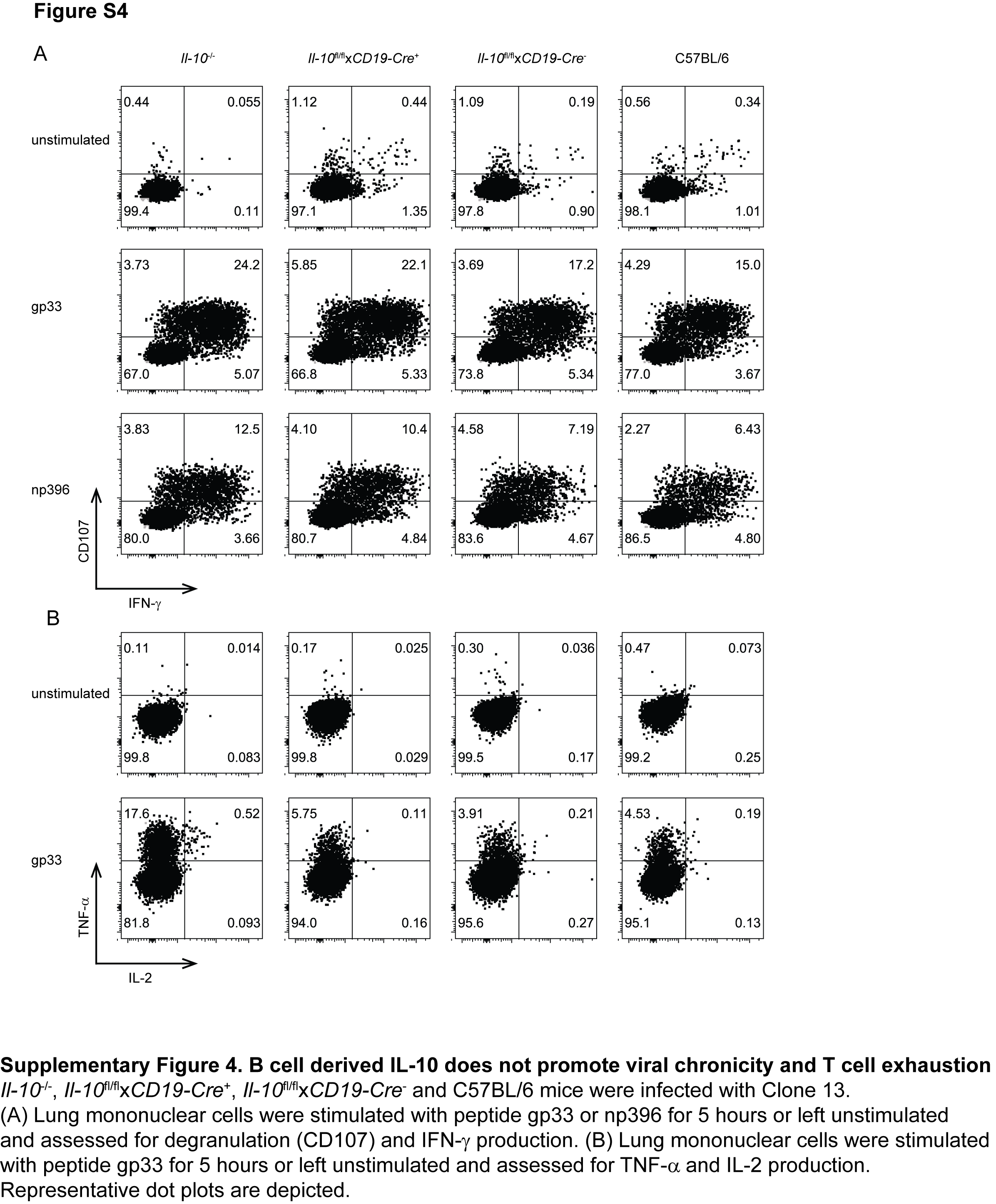

Supplement: Figure S4 — B cell derived IL-10 does not promote viral chronicity and T cell exhaustion. (TIF) [file ppat.1003735.s004.tif]

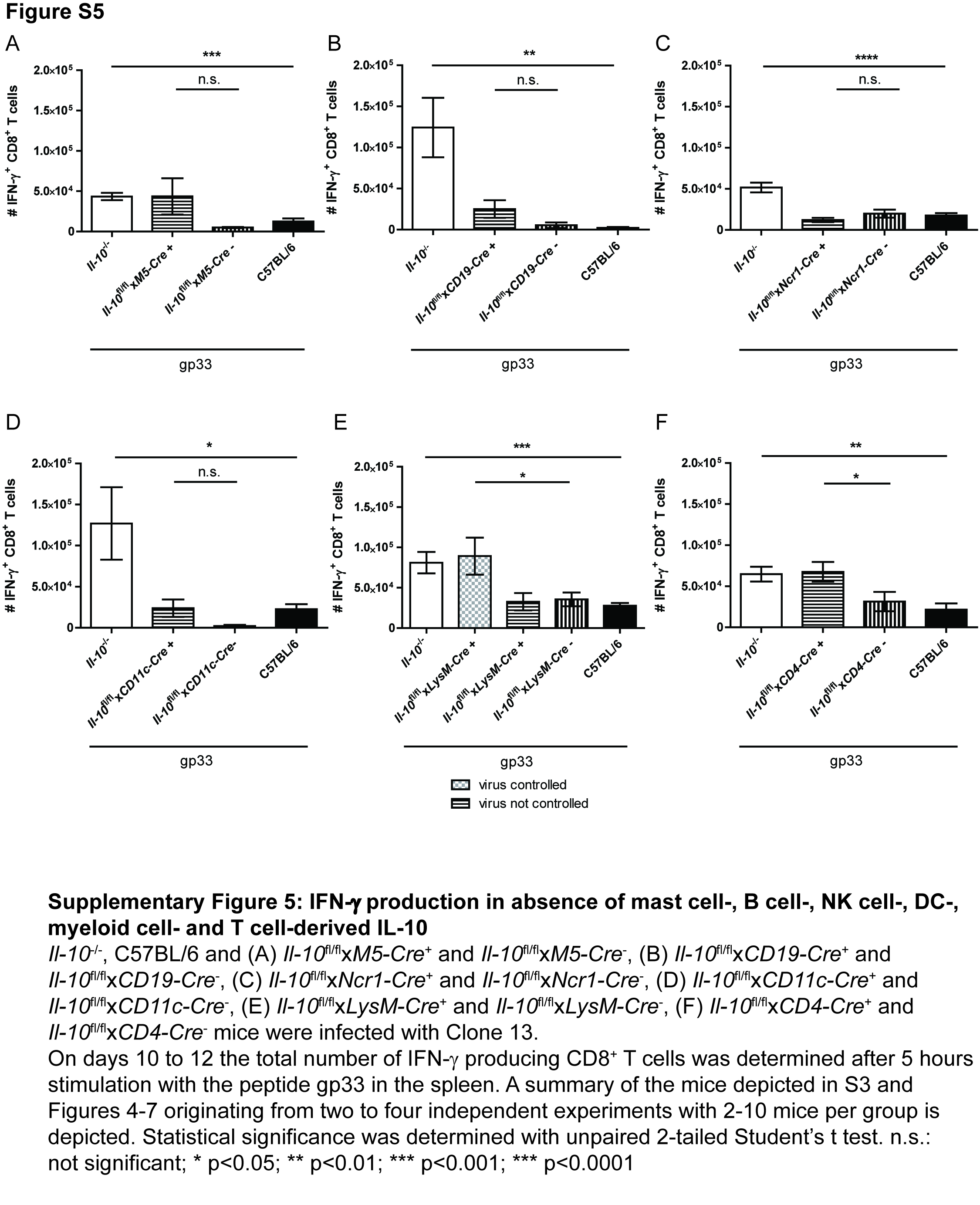

Supplement: Figure S5 — IFN-γ production in absence of mast cell-, B cell-, NK cell-, DC-, myeloid cell- and T cell-derived IL-10. (TIF) [file ppat.1003735.s005.tif]

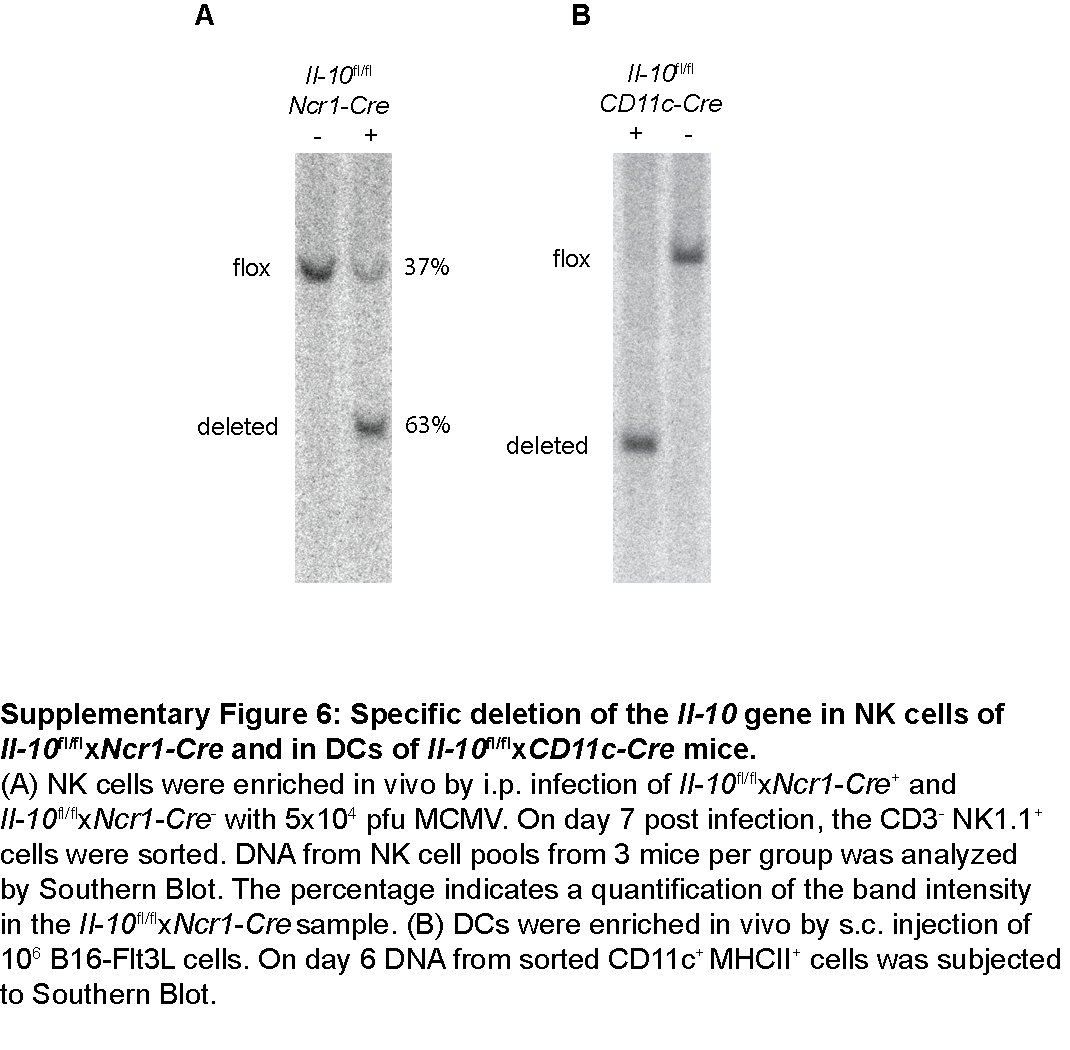

Supplement: Figure S6 — Specific deletion of the Il-10 gene in NK cells of Il-10 fl/flxNcr1-Cre and in DCs of Il-10 fl/flxCD11c-Cre mice. (TIF) [file ppat.1003735.s006.tif]

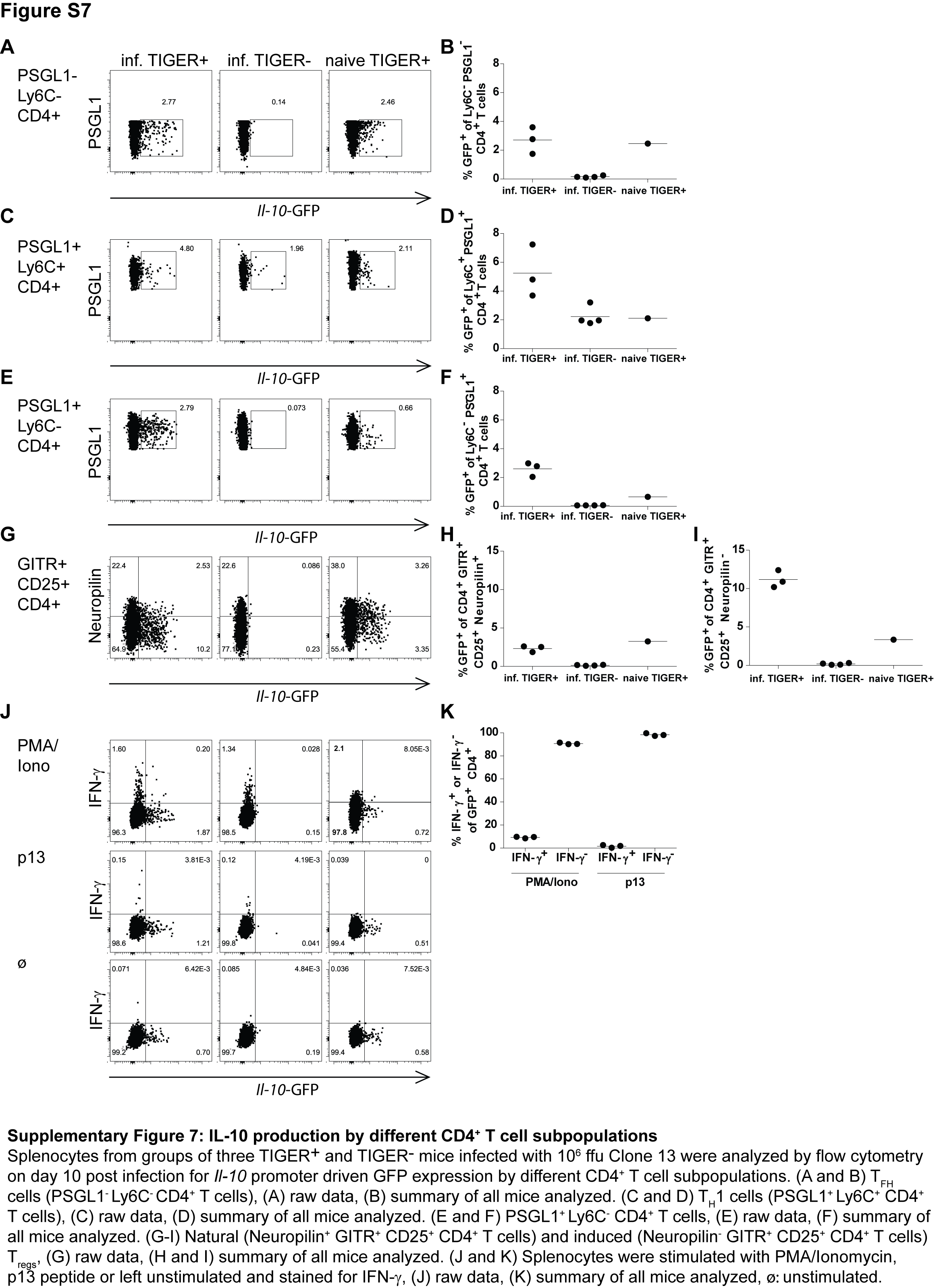

Supplement: Figure S7 — IL-10 production by different CD4+ T cell subpopulations. (TIF) [file ppat.1003735.s007.tif]
